# T-DNAreader: fast and precise identification of T-DNA insertion sites in plant genomes using RNA sequencing data

**DOI:** 10.1186/s13059-025-03655-x

**Published:** 2025-07-10

**Authors:** Hongwoo Lee, Pil Joon Seo

**Affiliations:** 1 Department of Chemistry, Seoul National University, Seoul, 08826 Korea; 2 Plant Genomics and Breeding Institute, Seoul National University, Seoul, 08826 Korea

**Keywords:** T-DNA, T-DNA insertion site, Unannotated T-DNA, RNA-seq, Whole-genome sequencing (WGS), ChIP-seq, Bioinformatic tool

## Abstract

*Agrobacterium*-mediated plant transformation, which enables the delivery of DNA using transfer DNA (T-DNA) binary vectors, is an essential technique in plant research. T-DNAs randomly integrate into the host genome, and multiple T-DNAs can sometimes integrate during a single transformation, necessitating the development of tools to elucidate the T-DNA insertion sites (TISs). Here, we present T-DNAreader, which identifies TISs from RNA sequencing data with high precision, sensitivity, and speed, outperforming existing tools. T-DNAreader enables the efficient and reliable identification of TISs within transcribed regions and standardizes the characterization of T-DNA-containing transgenic plants, which can be expanded to various organisms containing T-DNA.

**Supplementary Information:**

The online version contains supplementary material available at 10.1186/s13059-025-03655-x.

## Background

*Agrobacterium tumefaciens* is a phytopathogenic soil bacterium that infects wounded plant tissues and causes crown gall disease by delivering transfer DNA (T-DNA) into host plant cells through a bacterial type IV secretion system [1, 2]. Harnessing this capability, *Agrobacterium* was engineered as a plant transformation tool to deliver a gene of interest (GOI) that is inserted between the left border (LB) and right border (RB) of the T-DNA region, replacing the crown gall oncogenes [1, 3, 4]. *Agrobacterium*-mediated genetic transformation has been successfully used in many plant species, such as *Arabidopsis thaliana*, rice (*Oryza sativa*), maize (*Zea mays*), and tomato (*Solanum lycopersicum*) [5–8]. DNA sequences, regardless of their origin, have been efficiently introduced into host plants, enabling a wide range of genetic engineering.

In addition to delivering GOIs, T-DNAs have been used for insertional mutagenesis because the T-DNAs randomly integrate into host plant genomes. Indeed, for the model plant *Arabidopsis*, more than 700,000 *Agrobacterium*-mediated T-DNA insertion mutant lines have been generated, among which ~ 300,000 have been annotated using targeted T-DNA sequencing [9–13]. These publicly available T-DNA insertion mutants are invaluable resources for investigating gene functions, enabling significant advances in plant genetics and biological research. However, multiple T-DNAs can integrate during a single transformation, potentially causing background mutations that confound the analysis of mutant phenotypes.

In addition to causing background mutations, the presence of multiple T-DNA insertions can induce unwanted side effects in host plant genomes, such as large chromosomal rearrangements [14–26], which are likely associated with double-strand break repair processes [27, 28]. Chromosomal translocations/rearrangements have been observed in ~ 19% of SALK T-DNA lines [17] and 10% of GABI-Kat lines through Oxford Nanopore Technologies long-read sequencing [22]. Such chromosomal rearrangements not only disrupt gene structures at the DNA breakpoints, but also induce the misregulation of adjacent genes, possibly due to changes in the local chromatin/epigenetic environments [29, 30], necessitating efficient approaches to precisely identify T-DNA insertion sites (TISs) at a global scale.

The accurate identification of TISs in the genomes of transgenic plants is essential for studying the effect of genetic manipulations without unknown confounding factors. TISs have been traditionally identified by sequencing flanking sequence tags (FSTs) containing T-DNA border sequences and adjacent genomic regions using techniques such as thermal asymmetric interlaced polymerase chain reaction (TAIL-PCR) and adapter-ligated PCR [31–33]. However, these methods preferentially detect a single FST per line, even when multiple TISs are present in the genome [33]. Furthermore, despite the improved sensitivity of modified TAIL-PCR in the identification of TISs, the T-DNA frequently becomes truncated prior to genomic integration, making FST detection challenging [25–27, 34]. Indeed, although most publicly available T-DNA insertion lines have been reported to contain a single insertion, the average number of T-DNA insertions per line has been estimated to be ~ 1.5 [35]. Consistent with this, Oxford Nanopore Technologies long-read sequencing identified 11 previously unannotated T-DNA insertions from 14 GABI-Kat T-DNA insertion lines, raising the possibility of an underestimated number of T-DNA insertions in transgenic plants [22].

Several bioinformatic tools have been developed to identify the positions of TISs in host plant genomes [36–38]. Methodological frameworks CONTRAILS [36] and TDNAscan [37] are often used to define TISs by detecting discordantly mapped read pairs, in which one read aligns to the reference genome and the other aligns to the T-DNA region, but these tools have only limited application to paired-end sequencing data. Recently, T-LOC has been developed to capture soft-clipped reads, which are partially aligned to the reference genome and contain a portion of unmapped sequences [38]. Although all these tools often have been used to identify TISs, they require whole-genome sequencing (WGS) data, which are not routinely produced for plant genetic studies and are inefficient to regularly produce for TIS identification alone.

Therefore, there is a growing need to develop a bioinformatics tool for TIS identification using types of sequencing data that are more frequently generated during plant-related studies, such as RNA sequencing (RNA-seq) transcriptomic data. Although transcriptomic data cover only the transcribed regions, genes and transposable elements together represent a substantial portion of the genome in small-genome species with high gene density. For instance, up to 55% of the *Arabidopsis* genome, based on the Araport11 annotation, can be expressed as RNA transcripts [39]. Furthermore, the identification of TISs using RNA-seq data should be useful, given that most studies focus on the effect of T-DNAs inserted in transcribed regions or in intergenic regions that influence nearby transcription. Existing TIS-searching tools are not optimized for use with RNA-seq data that exhibit biased coverage of transcribed regions, high transcript mutation rates, and chimeric transcripts resulting from trans-splicing events. Here, we developed T-DNAreader, a tool designed to identify TISs from RNA-seq data, and demonstrated its high sensitivity, precision, and speed for TIS identification, outperforming other existing TIS-searching tools. T-DNAreader identified > 80% of annotated TISs and uncovered previously unannotated TISs using RNA-seq data. Furthermore, T-DNAreader is compatible with various types of sequencing data, including WGS data, and can be widely used to check for unexpected genetic changes caused by *Agrobacterium*-mediated transformation.

## Results

### Development of T-DNAreader framework

**Fig. 1:**
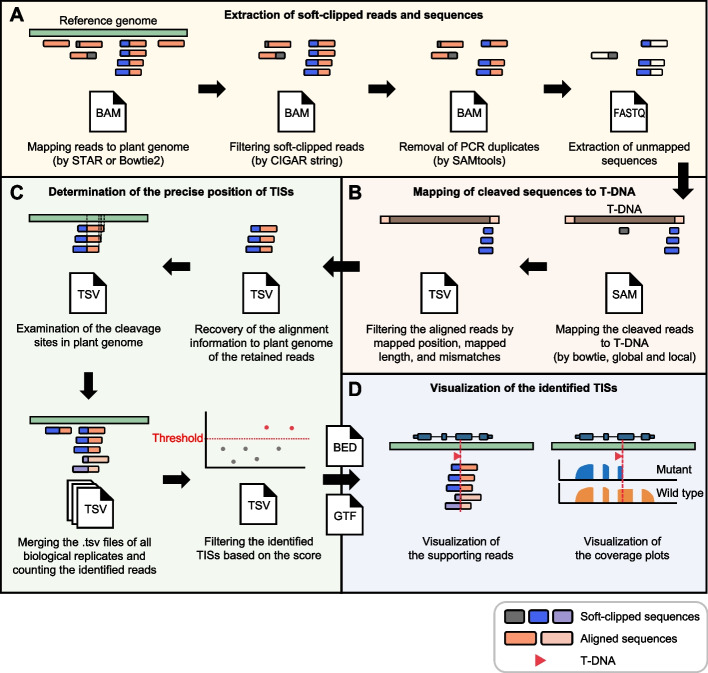
Workflow of T-DNAreader. The T-DNAreader pipeline consists of four processes. **A** FASTQ files are aligned to the reference genome, and then soft-clipped reads are collected from aligned BAM files based on the CIGAR string “S”. After removing duplicate reads, new FASTQ files containing the unmapped portions of the soft-clipped reads are then generated (cleaved FASTQ files). **B** The cleaved FASTQ files are mapped to the provided T-DNA sequences. Reads successfully mapped to the T-DNA sequences are filtered based on (1) their alignment position within the T-DNA, (2) the length of mapped sequences, and (3) the number of mismatches allowed. **C** The original alignment of the retained reads to the plant genome is restored, and their exact cleavage sites are identified. The information from all biological replicates is merged, and a confidence score is calculated for each candidate TIS. The TISs with scores above a defined threshold are retained. **D** T-DNAreader generates visualizations for each identified TIS, including supporting reads and coverage plots. The genomic positions and orientations of the T-DNA insertions are shown with the corresponding genomic annotations. In **A**–**D**, T-DNA and genomic segments of chimeric reads, and soft-clipped reads originating from different replicates are displayed in distinct colors

The growing need for the efficient and effective identification of TISs [40, 41] and the widespread availability of RNA-seq data motivated us to develop T-DNAreader, which is optimized to identify TISs from RNA-seq datasets. The T-DNAreader pipeline begins by aligning reads using the RNA-seq aligner STAR [42] or other local sequencing aligners including Bowtie2 [43] and BWA [44] (Fig. 1A). Then, this pipeline extracts soft-clipped reads from the alignment BAM files. After PCR duplicates are removed, the unmapped sequences of the soft-clipped reads are extracted to create cleaved FASTQ files. The cleaved FASTQ files are used to align with the T-DNA sequences using the Bowtie2 aligner (Fig. 1B). Small insertions or deletions are occasionally observed at T-DNA junctions [27, 41, 45, 46], so both end-to-end and local alignments are conducted. The T-DNA-mapped reads are filtered, based on (1) their alignment position within the T-DNA, (2) the length of the mapped sequences, and (3) the number of mismatches allowed. Because T-DNA insertions occur mainly through the LB and RB regions, the reads mapped close to the T-DNA borders (e.g., < 200 bp) are filtered with a threshold minimum mapped sequence length (e.g., ≥ 18 bp). By contrast, the reads mapped to the regions far from the T-DNA borders (e.g., ≥ 200 bp) are subjected to a strict threshold minimum mapped sequence length (e.g., ≥ 30 bp) to minimize false positives.

Subsequently, the original genomic positions aligned with the retained reads are retrieved to determine the precise TISs (Fig. 1C). Chimeric reads containing T-DNA insertion junctions are collected from all biological replicates, and a weighted sum of the confidence scores from supporting reads is calculated for each TIS. Predicted TISs with scores exceeding a defined threshold are classified as true TISs. Finally, T-DNAreader visualizes these sites along with their read alignments and coverage plots, enabling intuitive interpretation of the insertion context and its potential consequences (Fig. 1D). The whole implementation process of T-DNAreader can be parallelized, enabling a stringent and fast analysis for TIS identification.

### T-DNAreader identifies TISs from RNA-seq data with high sensitivity and precision

To evaluate the performance of T-DNAreader in TIS identification from RNA-seq data, we applied our tool to 57 public RNA-seq datasets generated from many *Arabidopsis* mutants suspected to have T-DNA insertions in genic regions (58 SALK, 11 GABI, and three SAIL lines) (Additional file 2: Table S1) [47–87]. For the TIS identification, we utilized the T-DNA sequences of the pROK2 (SALK), pAC161 (GABI), and pCSA110 or pDAP101 (SAIL) plasmids.

**Fig. 2:**
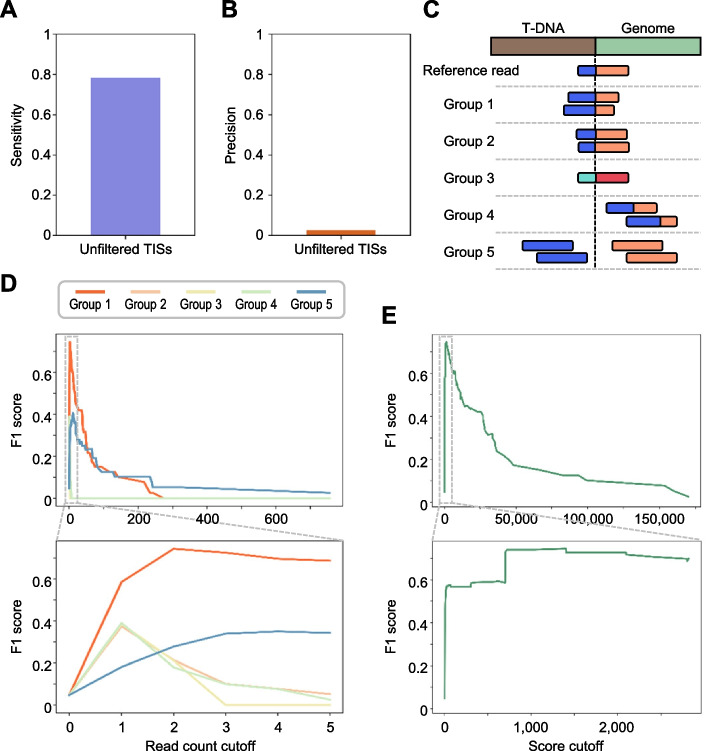
Estimation of confidence scores for TISs predicted by T-DNAreader. **A**–**B** Sensitivity (**A**) and precision (**B**) of T-DNAreader for detecting annotated TISs in 72 *Arabidopsis* T-DNA mutants without applying a score threshold. **C** The five categories of chimeric or discordant reads used by T-DNAreader to calculate confidence scores. Sequences aligned to T-DNA are shown in blue or light blue; sequences aligned to the genome are shown in red or orange. The light-blue or red indicates chimeric reads derived from a different biological replicate. **D**–**E** F1-score curve showing T-DNAreader performance as a function of the minimum number of supporting reads (**D**) and the confidence score threshold (**E**). The confidence score is calculated as the weighted sum of supporting reads from the five categories

A total of 2533 candidate TISs were initially predicted without applying any score threshold (Fig. 2A, B). This low precision likely stems from RNA-seq-specific issues, such as intermittent sequencing errors and spontaneous transcript fusion artifacts, underscoring the need for a robust quality check method to distinguish true TISs [88, 89]. To address this, we introduced a scoring system based on the number and types of supporting reads at each predicted TIS (Fig. 2C). When a chimeric read is detected at a genomic position, it is designated as the reference read. Other additional supporting reads within surrounding region are then classified into five categories based on their relationship to the reference read: (1) chimeric reads with different length ratios of T-DNA to genome sequence, (2) reads differing by only one base from the reference, (3) reads identical to the reference but derived from different biological replicates, (4) chimeric reads with nearly but non-identical junction sites, and (5) discordant read pairs where one maps to T-DNA and the other to the adjacent region of the predicted junction.

We evaluated F1 scores across a range of read-count thresholds for each group and found that Group 1 supporting reads contributed most significantly, as expected (Fig. 2D). Thus, we assigned default weights of 700, 300, 300, 1, and 1 to Groups 1–5, respectively, for final score calculation. The F1-optimized cutoff approximated ~ 1000, suggesting that a true TIS typically requires support from at least three independent chimeric reads—commonly one reference read, one Group 1 read, one additional read from Groups 1–3 (Fig. 2E). Since the reads from Groups 4 and 5 received minimal weight, overall performance remained unchanged regardless of whether discordant pairs were included (Additional file 1: Fig. S1). Given the additional processing time required to extract discordant pairs, their use is optional. For downstream analyses in *Arabidopsis*, we set the default score threshold to 1000 and excluded discordant pairs.

**Fig. 3:**
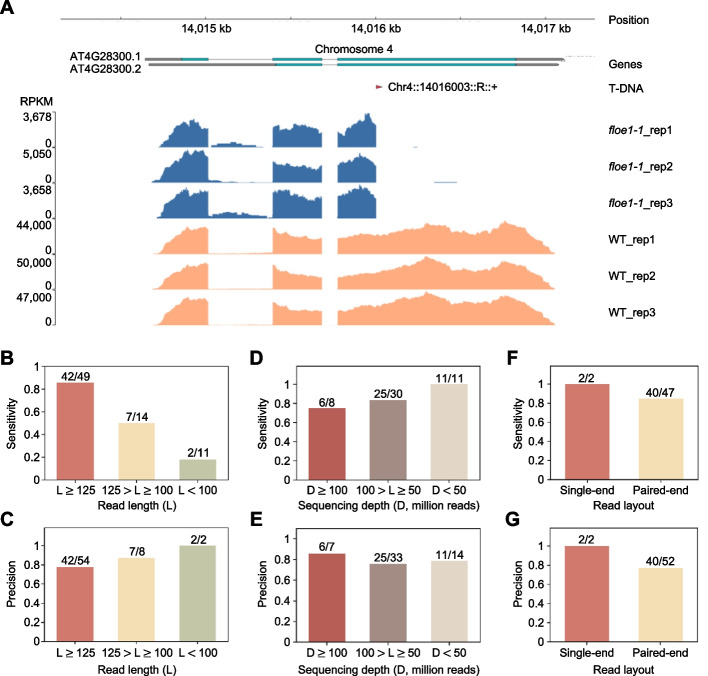
Analysis of public *Arabidopsis* RNA-seq datasets using T-DNAreader. **A** An example region of the *FLOE1* locus predicted to harbor a T-DNA insertion by T-DNAreader. The TIS and RNA-seq coverage plots are visualized by T-DNAreader. The RNA-seq coverage was calculated as the number of reads per kilobase of transcript per million mapped reads (RPKM). The red triangle indicates the predicted TIS, including its orientation. The T-DNA label indicates the chromosome, insertion site, relative configuration to the reference genome (R: REF–T-DNA; L: T-DNA–REF), and T-DNA orientation (+ or −). **B**–**G** The performance of T-DNAreader according to read length (**B** and **C**), sequencing depth (**D** and **E**), and read layout (**F** and **G**), with the sensitivity of T-DNAreader displayed in (**B**, **D**, **F**) and its precision in (**C**, **E**, **G**). In (**D**–**G**), RNA-seq data with read lengths of 125 bp or longer were used for comparison

By applying our stringent scoring system, no TISs were detected by T-DNAreader in the 40 RNA-seq datasets derived from wild-type *Arabidopsis* (Col ecotype) as a negative control (Additional file 2: Table S2); however, T-DNAreader identified 64 TISs. Of these, 51 matched the original annotation of the TISs, while 13 TISs that were not annotated in the T-DNA mutant databases were newly identified (Additional file 2: Table S2). In support, T-DNAreader showed that RNA-seq coverage was interrupted at the predicted TIS, which was supported by multiple chimeric reads, as shown in the *floe1-1* mutant (Fig. 3A; Additional file 1: Fig. S2). T-DNAreader found some mutants with more than one TIS within a single gene, as shown in the *plt1-21 plt2-21* double and *nlp7-1* mutants (Additional file 2: Table S2). Given that G/T or A/G sequences were frequently observed at these T-DNA junctions, it is likely that the multiple TISs originate from a single T-DNA integration due to mis-splicing events caused by the T-DNA insertion (Additional file 1: Fig. S3).

The RNA datasets have variable read layouts (single-end and paired-end reads), sequencing depths, and read lengths (usually ranging from 50 to 151 bp), so we examined the effect of these factors on T-DNAreader-assisted TIS identification. T-DNAreader showed the highest sensitivity, 0.86 (42 sites matched with the original annotation/49 total annotated sites), along with a reasonable precision of 0.78 (42 sites matched with the original annotation/54 total identified sites) when using relatively long read lengths (≥ 125 bp), which is the most common lengths used in recent NGS analyses (Fig. 3B, C). Considering that the precision value was estimated based only on the annotated TISs, the real precision rate is likely to be higher. For RNA-seq datasets with long read lengths (≥ 125 bp), T-DNAreader could identify TISs even with low sequencing depths of under 50 million reads or using single-end reads with high sensitivity, indicating that the read length is the most critical factor for the T-DNAreader performance (Fig. 3D–G). Overall, these results indicate that T-DNAreader is compatible with RNA-seq data in identifying TISs with reasonable sensitivity and precision.

### Identification of TISs in transgenic overexpression lines using T-DNAreader

The TIS identification strategy of T-DNAreader is not limited to T-DNA insertional mutants; it can be extended to detect TISs in transgenic plants expressing any GOI. As a proof of concept, we applied T-DNAreader to identify TISs in transgenic *Arabidopsis* plants overexpressing several different GOIs. RNA-seq datasets from 11 transgenic overexpression lines were analyzed using T-DNAreader with their respective backbone vector sequences (Additional file 2: Table S1, S3) [65, 90–97]. In cases where the T-DNA contained an endogenous gene(s), the corresponding genomic coordinates were defined as blacklist regions. Notably, T-DNAreader identified 14 TISs from eight transgenic lines, 10 of which accompanied mis-spliced, truncated, or extended transcripts potentially due to the T-DNA insertion (Additional file 1: Fig. S4 and Additional file 2: Table S3), suggesting that T-DNAreader can be used to identify TISs in transgenic plants and to predict potential genomic perturbance caused by T-DNA insertions.

### T-DNAreader outperforms conventional T-DNA searching tools using RNA-seq data

**Fig. 4:**
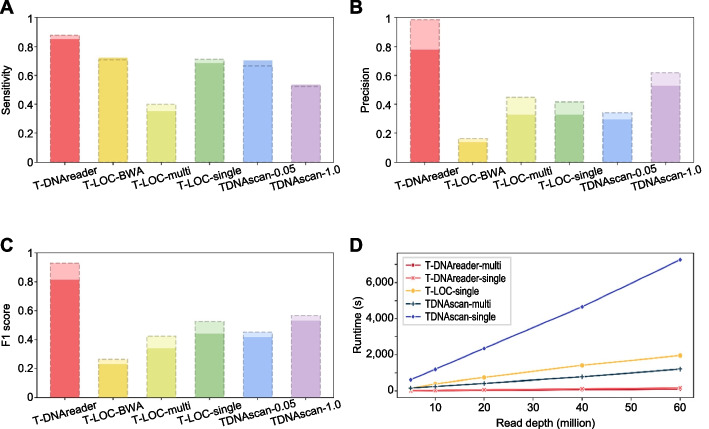
Benchmarks of T-DNAreader against T-LOC and TDNAscan. **A–C** Comparison of sensitivity (**A**), precision (**B**), and F1 score (**C**) between T-DNAreader, T-LOC, and TDNAscan. T-LOC was implemented with BWA aligner (T-LOC-BWA), STAR aligner with a single replicate of control data (T-LOC-single), or STAR aligner with multiple replicates of control data (T-LOC-multi). Tool performance was evaluated using both the original annotations (solid colors) and RNA-seq signal disruptions (lighter colors with dashed outlines). The results of TDNAscan were filtered with insertion frequency thresholds of 0.05 (TDNAscan-0.05) and 1.0 (TDNAscan-1.0). **D** Runtime comparison between T-DNAreader, T-LOC, and TDNAscan. T-DNAreader was implemented with a single thread (T-DNAreader-single) and two threads (T-DNAreader-multi). For T-LOC, the runtime was measured using T-LOC-single with single control data. TDNAscan was implemented with a single thread (TDNAscan-single) and with eight threads (TDNAscan-multi). Subsampled data from *plt1-21 plt2-21* RNA-seq data (SRR14850977) were used for the runtime benchmark

No TIS identification tools have previously been developed to utilize RNA-seq data; therefore, we compared the power of T-DNAreader with those of TDNAscan [37] and T-LOC [38], which were originally designed to identify TISs using WGS data. Given that TDNAscan is compatible only with paired-end reads, 43 paired-end RNA-seq datasets using 53 T-DNA mutants were used for the comparison. TDNAscan was implemented with two different cutoff values of the insertion frequencies: 0.05 (TDNAscan-0.05) and 1.0 (TDNAscan-1.0). In addition, T-LOC was implemented with different aligners, including BWA DNA aligner (T-LOC-BWA) as well as STAR RNA-seq aligner using a single control replicate (T-LOC-single) and using multiple control replicates (T-LOC-multi). When we compared the sensitivity and precision based on the original annotation, T-DNAreader identified 46 TISs out of the 54 annotated TISs, exhibiting the highest sensitivity (0.85) among the tools tested (Fig. 4A). Furthermore, T-DNAreader exhibited high precision of 0.78, but T-LOC and TDNAscan showed low precision with a range from 0.14 to 0.53 (Fig. 4A, B; Additional file 2: Table S4, S5). The F1 score, representing the harmonic mean of precision and sensitivity, also showed T-DNAreader to be the most effective tool for identifying TISs from RNA-seq data (Fig. 4C). To address limitations of using only annotated TISs, we also evaluated tool performance based on RNA-seq coverage alterations at the predicted sites. As a result, the precision of T-DNAreader markedly increased to 0.98 (Fig. 4B). In contrast, most false-positive TISs predicted by other tools showed no detectable RNA-seq coverage disruption (Additional file 2: Table S4, S5) and corresponded to a zero score in T-DNAreader (Additional file 1: Fig. S5), highlighting the value of stringent thresholds and a robust scoring system in RNA-seq-based TIS identification.

We next benchmarked the runtime of these tools using randomly subsampled RNA-seq data produced from the *plt1-21 plt2-21* double mutant [69]. Because each tool requires different input formats and unique post-processing steps, we measured the runtime covering the core process that predicts TISs from the alignment files (BAM format) for a fair comparison. T-DNAreader and TDNAscan, which support multithreading, were implemented in multiple- (two threads for T-DNAreader; eight threads for TDNAscan) or single-thread mode. Both T-DNAreader-single and T-DNAreader-multi were substantially faster than TDNAscan and T-LOC: T-DNAreader-single was 41 × and 11 × faster than TDNAscan-single and T-LOC, respectively, when processing 60 million reads, whereas T-DNAreader-multi was 13 × and 21 × faster than TDNAscan-multi and T-LOC (Fig. 4D). T-DNAreader-multi processed 60 million reads within 2 min; extraction of discordant pairs added processing time but remained efficient (Additional file 1: Fig. S6A). Furthermore, although T-LOC exhibited extremely low processing speeds for TIS identification from some RNA-seq data, T-DNAreader maintained a high speed (Additional file 1: Fig. S6B). Overall, our benchmarking results demonstrate that T-DNAreader enables TIS identification with high sensitivity, high precision, and low computational time.

### T-DNAreader identifies previously uncharacterized TISs using RNA-seq data

**Fig. 5:**
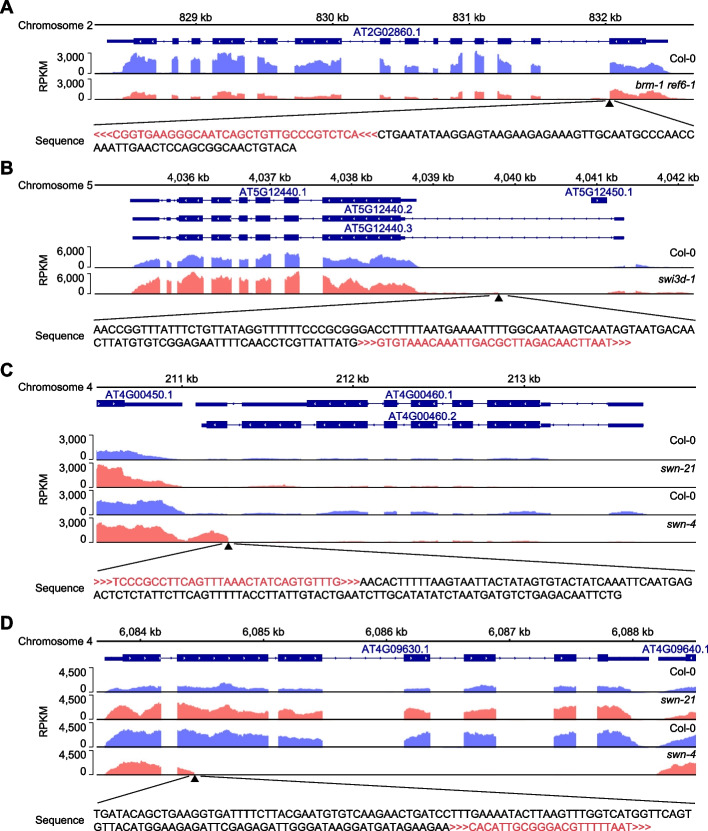
Misregulation of RNA splicing or gene transcription at the previously unannotated T-DNA insertion sites (TISs) identified using T-DNAreader. **A–D** RNA-seq coverage plots at the previously unannotated T-DNAreader-identified TISs in the wild type and T-DNA insertion mutants. The RNA-seq coverage was calculated as the number of reads per kilobase of transcript per million mapped reads (RPKM). The black triangles indicate the T-DNAreader-identified TISs. The T-DNA sequences are colored red, and the orientation of the T-DNA insertion is indicated

Given the high precision of TIS identification by T-DNAreader using RNA-seq data, we focused on 13 T-DNAreader-identified TISs that had not been previously annotated. T-DNAreader identified additional TISs within the intron regions of *SUCROSE TRANSPORTER 2* (*SUT2*, *AT2G02860*) in the *brm-1 ref6-1* double mutant, *ATC3H55* (*AT5G12440*) in the *swi3d-1* mutant, and *AT3G26100* in the *cmt3-11* mutant. These cases exhibited aberrant RNA-seq coverage at the TIS-containing intronic regions due to impaired RNA splicing caused by the T-DNA insertion (Fig. 5A, B; Additional file 1: Fig. S7A).

Moreover, an additional T-DNA insertion was located in the promoter region of the *EXOCYST SUBUNIT EXO70 FAMILY PROTEIN H4* (*EXO70H4*, *AT3G09520*) in the *rve5-2* mutant, leading to the overexpression of abnormal extended transcripts containing its promoter elements (Additional file 1: Fig. S7B). Similarly, in the *fry1-6 rdr1-1*, *jmj28-2*, and *nlp7-1* mutants, aberrant expression patterns were detected at the predicted TISs (Additional file 1: Fig. S7C-H). In addition, T-DNAreader identified two additional TISs at the *ROP GUANINE NUCLEOTIDE EXCHANGE FACTOR 3* (*ROPGEF3*, *AT4G00460*) and *AT4G09630* loci in the *swn-4* T-DNA insertion mutant (Additional file 2: Table S2). As expected, misregulated and truncated transcripts of *ROPGEF3* and *AT4G09630* were observed in the *swn-4* mutant, but not in the *swn-21* mutant, suggesting the presence of T-DNA in multiple regions of *swn-4* (Fig. 5C, D). Overall, 12 out of 13 predicted TISs, which were previously unannotated, showed clear evidence of aberrant RNA-seq signals are likely true positives.

**Fig. 6:**
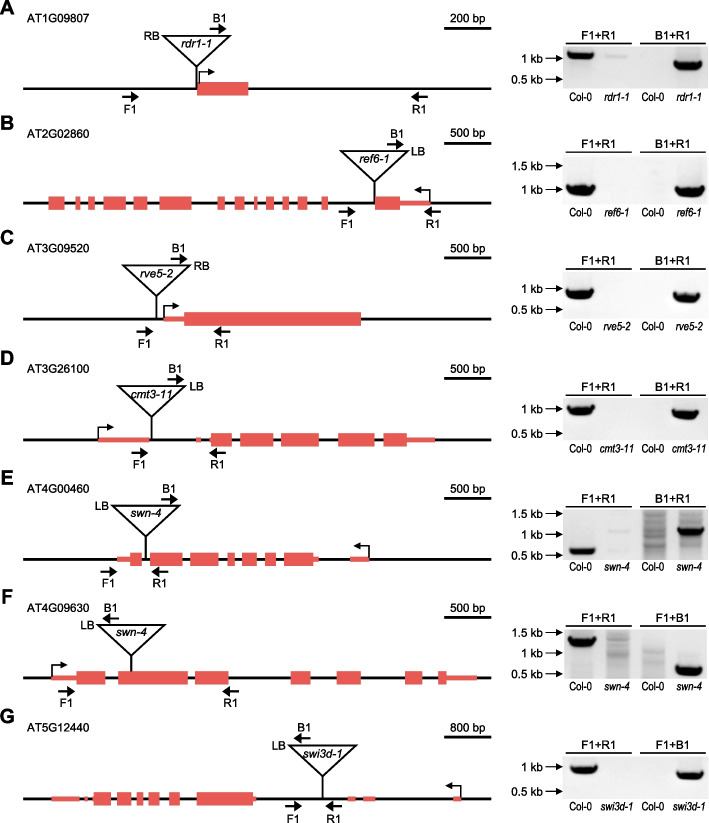
Validation of the previously unannotated T-DNA insertion sites (TISs) using PCR. **A–G** Previously unannotated TISs in seven loci (*AT1G09807*, *AT2G02860*, *AT3G09520*, *AT3G26100*, *AT4G00460*, *AT4G09630*, and *AT5G12440*) in *Arabidopsis* mutants were validated. Schematic diagrams for each gene illustrate the identified TISs, as well as the primers used for the PCR validation. The orientations and positions of the T-DNA insertions are also displayed. Primers F1 and R1 amplify the genomic regions flanking the TIS, and primer B1 binds to the inserted T-DNA. Primer pairs of F1-R1 and F1/R1-B1 were used for the PCR amplification

We used PCR-based genotyping to verify the TISs newly identified by T-DNAreader. To genotype the high-order mutants, we obtained the eight parental single mutants to determine the origin of the additional T-DNA and confirmed the T-DNA insertion for seven TISs (Fig. 6; see Discussion): Chimeric fragments composed of T-DNA and genomic sequences were amplified at the T-DNAreader-identified regions in those T-DNA insertion mutants (Fig. 6). The T-DNA border regions were also examined by Sanger sequencing, confirming the presence of the T-DNAreader-identified TISs at the predicted sites (Additional file 1: Fig. S8). Overall, our results demonstrate that T-DNAreader precisely identifies TISs and can be used to uncover previously unidentified TISs.

### Chromosomal rearrangement occurs between additional TISs

**Fig. 7:**
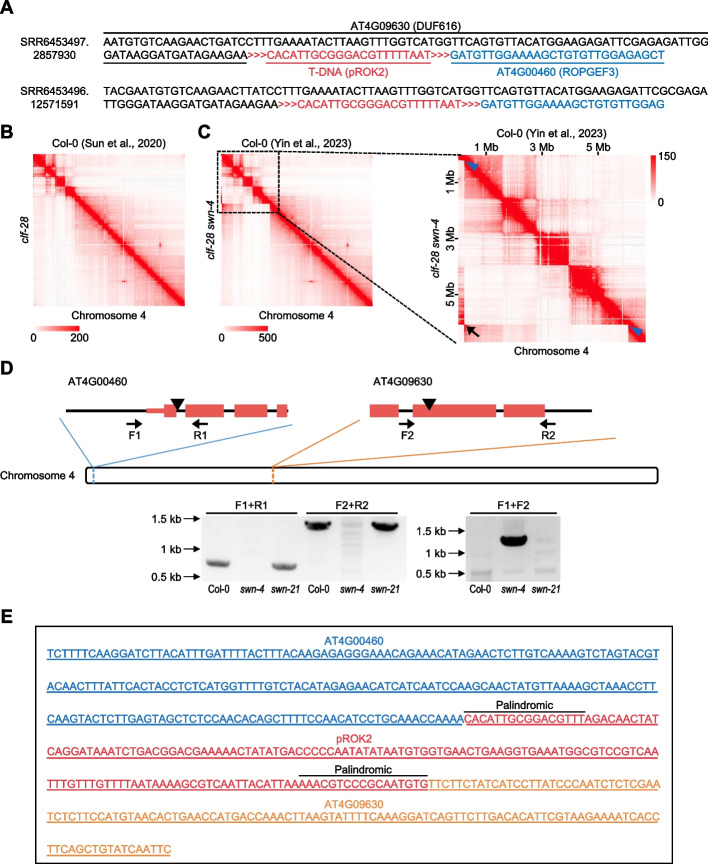
Chromosomal rearrangements between uncharacterized T-DNA insertion sites (TISs) in *swn-4. ***A** RNA-seq reads containing sequences from *ROPGEF3* (black), *AT4G09630* (blue), and T-DNA (red) in the *swn-4* mutant. **B** Knight-Ruiz (KR)–normalized Hi-C contact maps of chromosome 4 at 25-kb resolution for the wild type (Col-0) and the *clf-28* mutant. The upper-right triangle displays the Hi-C contact map of the wild type, and the lower-left triangle exhibits that of *clf-28*. **C** KR-normalized Hi-C contact maps of chromosome 4 in the wild type and* clf-28 swn-4 *mutant at 25- (left) and 10-kb (right) resolution. The upper-right triangle displays the contact map of Col-0, and the lower-left triangle exhibits that of *clf-28 swn-4*. The blue triangles indicate T-DNAreader-identified TISs at the *ROPGEF3* and *AT4G09630* loci. The ectopic contact frequencies between the *ROPGEF3* and *AT4G09630* loci are highlighted (black arrow). **D** Validation of the chromosomal rearrangement between the *ROPGEF3* and *AT4G09630* loci in the *swn-4* mutant. The schematic diagrams illustrate the identified TISs as well as the primers used for the PCR validation. The amplified PCR products from the wild-type and *swn-4* and *swn-21* mutant plants are displayed. **E** Sanger sequencing results of the amplified chimeric fragments at the inversion junction. The sequences derived from *ROPGEF3*, *AT4G09630*, and the inserted T-DNA are shown in blue, red, and orange, respectively

It has been reported that multiple T-DNA insertions sometimes induce unwanted side effects in the genome, such as chromosomal rearrangements [17, 19, 22, 41]. This raises the possibility that a mutant line with unexpected additional T-DNA insertions may have unanticipated genomic alterations. Surprisingly, we found multiple RNA-seq reads concurrently containing partial sequences of *ROPGEF3*, *AT4G09630*, and T-DNA in the *swn-4* mutant (Fig. 7A), suggesting that chromosomal rearrangement events may have occurred between the TISs at *ROPGEF3* and *AT4G09630*, which were newly identified by T-DNAreader.

**Fig. 8:**
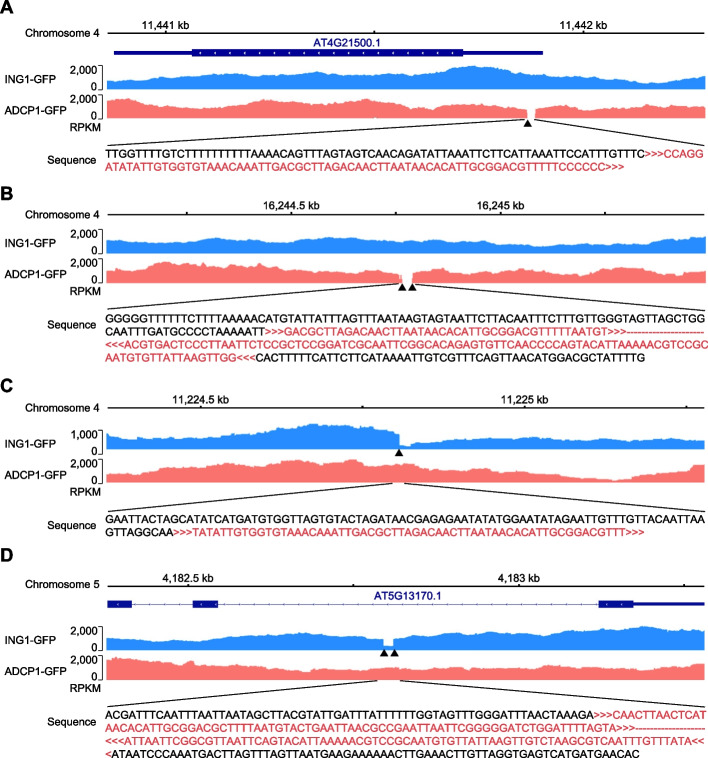
Examples of T-DNAreader-identified T-DNA insertion sites (TISs) from whole-genome sequencing (WGS) data. **A**–**D** WGS coverage plots at the T-DNAreader-identified TISs in *pADCP1::ADCP1-GFP* (**A** and **B**) and *pING1::ING1-GFP* (**C** and **D**) transgenic plants. The input data of ChIP-seq experiments were used for the analysis. The WGS coverage was calculated as the number of reads per kilobase of transcript per million mapped reads (RPKM). The black triangles indicate the T-DNAreader-identified TISs. The T-DNA sequences are colored red, and the orientation of the T-DNA insertion is indicated

To examine this hypothesis, we analyzed publicly available Hi-C (high-throughput chromatin conformation capture) datasets generated using wild-type, *clf-28*, and *clf-28 swn-4* mutant plants for the visualization of chromatin conformation [98–100]. Unlike for the wild type and *clf-28*, the *clf-28 swn-4* Hi-C contact map exhibited disrupted interaction signals at the *ROPGEF3* and *AT4G09630* loci (Fig. 7B, C). In addition, the high contact frequency between the *ROPGEF3* and *AT4G09630* loci (black arrow) indicated their spatial proximity in the genome. Furthermore, EagleC, a deep learning–based algorithm designed to identify chromosomal rearrangement events from Hi-C data [101], detected an inversion between the *ROPGEF3* and *AT4G09630* loci in the *clf-28 swn-4* plants, but not in the wild-type and *clf-28* plants, further supporting the presence of a large inversion event in chromosome 4 of the *swn-4* genome (Additional file 2: Table S6). We confirmed the chromosomal inversion in the *swn-4* mutant through a PCR analysis. Chimeric fragments comprising the *ROPGEF3* and *AT4G09630* sequences were amplified from the *swn-4* line, but not from the wild type and *swn-21* (Fig. 7D). Sanger sequencing of the PCR products also revealed that the *ROPGEF3* sequences were fused with inverted *AT4G09630* sequences, along with truncated T-DNA sequences flanked with palindromic repeats, suggesting that the chromosomal inversion occurred through the homologous recombination of T-DNA sequences (Fig. 7E). Taken together, the fast and accurate TIS identification gave a chance to detect secondary genomic damage induced by the T-DNA insertions.

### Identification of TISs from ChIP-seq data using T-DNAreader

Although T-DNAreader was initially designed for utilizing RNA-seq data, we wanted to determine whether it is also compatible with WGS data. We thus applied T-DNAreader to *Arabidopsis* chromatin immunoprecipitation sequencing (ChIP-seq) data, the most prevalent type of WGS data for functional studies using transgenic plants. We collected input datasets from ChIP-seq experiments, along with their respective backbone vector sequences [84, 102–106] (Additional file 2: Table S1, S7). T-DNAreader identified 15 TISs from six transgenic lines expressing endogenous genes fused with epitope tags and seven TISs from five T-DNA insertional mutant lines (Additional file 2: Table S7). All annotated TISs in the T-DNA insertional mutants were identified. Consistent with this, the NGS signals disappeared or dropped around the predicted TISs (20/22 TISs; 91%) (Fig. 8). These results indicate that T-DNAreader can also be used for TIS identification from WGS data.

### Application of T-DNAreader in various plant and fungal species

**Fig. 9:**
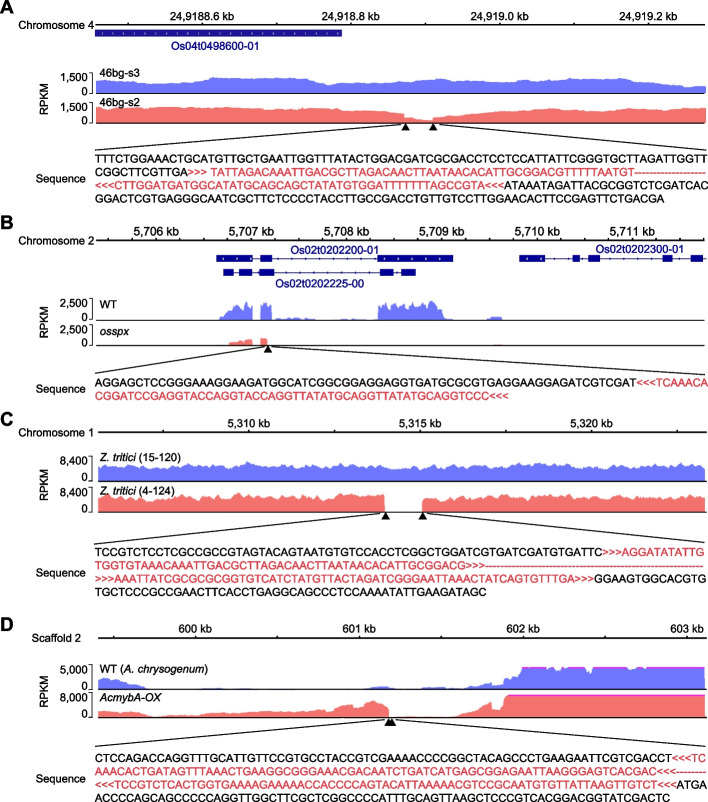
Examples of T-DNAreader-identified T-DNA insertion sites (TISs) in other species. **A**–**D** Coverage profiles at T-DNAreader-identified TISs in transgenic rice (**A** and **B**) and fungal species, *Z. tritici* (**C**) and *A. chrysogenum* (**D**). The WGS data (**A** and **C**) and RNA-seq data (**B** and **D**) were used for TIS identification. The coverage value is calculated as the number of reads per kilobase of transcript per million mapped reads (RPKM). The black triangles indicate the T-DNAreader-identified TISs. The T-DNA sequences are colored red, and the orientation of the T-DNA insertion is shown

To demonstrate the versatility and precision of T-DNAreader, we applied it to rice WGS data [38, 107]. From 48 transgenic rice plants, T-DNAreader identified 56 and 62 TISs using score thresholds of 1000 and 700, respectively (Additional file 2: Table S8). Notably, over 90% of predicted sites showed clear WGS coverage disruptions (53/56, 95% at the 1000 threshold; 56/62, 90% at the 700 threshold), highlighting the high precision of T-DNAreader (Fig. 9A; additional file 1: Fig. S9A; additional file 2: Table S8). We further applied T-DNAreader to RNA-seq datasets from 8 rice T-DNA insertion mutants and their corresponding wild-type controls, identifying 10 TISs—9 of which exhibited distinct RNA-seq coverage alterations [108–115] (Fig. 9B; additional file 2: Table S9).

To evaluate the broader applicability of T-DNAreader, we analyzed WGS and RNA-seq data from diverse species, including poplar, *Marchantia polymorpha* (*M. polymorpha*), *Zymoseptoria tritici* (*Z. tritici*), *Verticillium dahliae* (*V. dahliae*), and *Acremonium chrysogenum* (*A. chrysogenum*), whose genomes can be genetically transformed by *Agrobacterium* [116–124]. From eight *Z. tritici* WGS datasets, T-DNAreader identified 14 insertion sites and detected seven TISs from six fungal RNA-seq datasets, with most exhibiting clear coverage disruptions (Fig. 9C, D; additional file 1: Fig. S9; additional file 2: Table S10). Notably, T-DNAreader also successfully captured genome-integrated CRISPR cassettes in multiple *M. polymorpha* and poplar lines. In fungal strains engineered via targeted homologous recombination, all five intended events were confirmed without off-target integrations (Additional file 1: Fig. S9D; additional file 2: Table S10). These results demonstrate the reliability of TIS detection by T-DNAreader. Overall, T-DNAreader provides a versatile and robust platform for TIS identification across a wide range of organisms using both genomic and transcriptomic data.

## Discussion

Plant research heavily relies on *Agrobacterium*-mediated plant transformation. Using this technique, T-DNA is randomly integrated into host plant genomes, so researchers need to identify the TISs and any potential problems caused by plant transformation, such as additional T-DNA insertions and T-DNA-induced chromosomal rearrangements. Considering that the T-DNA insertion is particularly detrimental in genic regions and that it is not realistic to generate WGS data for each mutant, it is essential to develop an efficient and effective bioinformatics tool to detect TISs in transcribed regions using RNA-seq data.

Here, we developed a novel bioinformatics tool, T-DNAreader, that is highly optimized for TIS identification using RNA-seq data even with low sequencing depths (below 50 million reads) and single-end sequencing data, in the case of long read lengths (≥ 125 bp) (Fig. 3B–G). Since 150- and 151-bp paired-end sequencing has been adopted for the majority of RNA-seq analyses, T-DNAreader can be widely applied for TIS identification. Recent advances in Illumina sequencing technologies would support longer sequencing reads of up to 250–350 bp, which will further improve the T-DNAreader performance. A key feature of T-DNAreader is its quantitative scoring system, which evaluates the confidence of each predicted TIS and allows users to customize score thresholds to suit specific needs. T-DNAreader outperformed the existing TIS-identifying tools in terms of sensitivity, precision, and speed, demonstrating that it is the most effective tool for identifying TISs from RNA-seq data. It should also be noted that although T-DNAreader was originally designed to use RNA-seq data, it is also compatible with WGS data; thus, T-DNAreader can serve as a comprehensive tool to detect TISs across a wide range of transgenic organisms using various types of Illumina-based NGS data.

Not all TISs have been characterized due to the low sensitivity of the FST capture method [33], and many T-DNA-containing transgenic/mutant plants occasionally contain unexpected additional copies of T-DNA (Additional file 2: Table S2, S7). Multiple TISs can potentially cause unwanted side effects, such as the misregulation of RNA splicing and neighboring gene expression, as well as large chromosomal rearrangements, necessitating the careful examination of the numbers and positions of the T-DNA insertions. The high reliability of T-DNAreader enabled us to identify previously unannotated TISs in publicly available mutants from their RNA-seq data, making this software superior to T-LOC and TDNAscan.

Although we were unable to experimentally validate the T-DNAreader-identified TISs in the *atxr6-1* and *fry1-6* mutants, likely due to the segregation of unannotated T-DNA insertions over successive generations, 63 out of 64 predicted TISs exhibited abnormal transcript profiles near the identified TISs (Fig. 4B; additional file 2: Table S2). In contrast, most of the new TISs identified by T-LOC and TDNAscan were found in only a single replicate of RNA-seq data without corresponding changes in RNA-seq coverage signals (Additional file 2: Table S4, S5). This indicates the relevance of T-DNAreader in identifying unexpected TISs. We therefore propose that T-DNAreader can be widely used to verify transgenic plant materials with minimal genomic perturbance caused by T-DNA insertions using easily accessible NGS data.

Although T-DNAreader successfully identified TISs from RNA-seq data, it is important to remain cautious when using these datasets. Several T-DNA mutant lines, exemplified by the *plt1-21 plt2-21* and *nlp7-1* mutants, exhibit mis-splicing events, making it difficult to extract the precise TISs (Additional file 1: Fig. S3). Additionally, T-DNA insertions in the promoter and 5′-untranslated regions occasionally result in the loss of gene expression, as observed in *ctl1-2* and *klu-1*, making it impossible to identify TISs in genic regions without the corresponding sequencing reads (Additional file 1: Fig. S9). Despite these limitations, compatibility of T-DNAreader with diverse NGS datasets, including RNA-seq, ChIP-seq, and WGS, enables users to integrate evidence across experiments for robust and comprehensive TIS identification.

## Conclusions

There are growing concerns about the risk of incorrect analysis when using T-DNA insertional transgenic and mutant plants [40, 41]. Minimal guidelines have been proposed to prevent misunderstandings when using T-DNA-containing plants: (1) genotyping and sequencing using T-DNA border regions, (2) segregation analysis by outcrossing to the wild type, (3) phenotypic analysis of F_2_ individuals obtained from the outcross, (4) genetic complementation with a functional construct, and (5) phenotypic analysis with at least two different independent plant lines [40]. Along with these validations, T-DNAreader can provide additional invaluable information about the unintended effects of T-DNA insertions, preventing the misinterpretation of results from the corresponding plant materials.

## Methods

### Development of T-DNAreader

T-DNAreader takes raw or adapter-trimmed FASTQ files as input. During alignment, the minimum required length for soft-clipped segments is adjusted according to read length to maximize the recovery of informative reads. Paired-end reads are aligned independently, ensuring discordant pairs are captured.

From the alignment files, soft-clipped reads containing the CIGAR string “S” are extracted, and PCR duplicates are subsequently removed using the markdup function of SAMtools [125] to reduce false positives and accelerate processing speed. The unmapped sequences of these soft-clipped reads with their sequencing quality scores are collected to create new cleaved FASTQ files in cases where the length of the unmapped sequences is greater than the cutoff (e.g., ≥ 18 bp, “-l1” option). The reads containing a high proportion (0.8) of poly(N) within the genomic alignment part are excluded to reduce the false positives. The cleaved reads are aligned to the provided T-DNA sequences using Bowtie2 in both end-to-end and local modes [43]. Reads aligned to the T-DNA sequences are filtered based on their alignment positions within the T-DNA, the length of the mapped sequences, and the number of mismatches allowed. Reads mapped close to the T-DNA borders (e.g., < 200 bp, “-d” option) are filtered using a mild threshold of the minimum length of the mapped sequence (e.g., ≥ 18 bp, “-l1” option). In contrast, reads mapped to regions far from the T-DNA borders (e.g., ≥ 200 bp, “-d” option) are subjected to a stricter threshold for the minimum length of the mapped sequence (e.g., ≥ 30 bp, “-l2” option). Mapped reads with more than one mismatch are discarded (“-m” option).

Subsequently, the original genomic alignment positions of the retained reads are retrieved to determine the precise genomic positions of the integration sites. All chimeric reads identified across the biological replicates are collected, and a score for each TIS is calculated using a weighted sum of five groups of supporting reads (Fig. 2C). TISs with scores above a defined threshold are defined as true TISs. Genes overlapping the identified TISs are annotated, while blacklist regions are excluded using the intersect function of DeepTools [126]. Finally, T-DNAreader generates visualizations for each identified TISs, showing its supporting reads and coverage profiles, powered by bamCoverage and pyGenomeTrack of DeepTools [126].

For all analyses performed in this study, T-DNAreader was implemented using the default parameters except for the “-r” parameter, which was set as follows: 0.67 for reads < 100 bp, 0.5 for reads between 100 and 125 bp, and 0.33 for reads between 125 and 151 bp, to adjust the minimum soft-clip length. The “--paired” option was used for paired-end sequencing. The threshold of 1000 was applied by default, except for rice RNA-seq (2000). Additionally, regions encoding certain chloroplast genes (e.g., rbcS), which are prone to misidentification due to their involvement in vector construction, were blacklisted (Additional file 2: Table S11). Additionally, in the analyses of transgenic overexpression and tagging lines, regions encompassing the target corresponding genes and their 3-kb flanking sequences were also specified as blacklist regions.

### RNA-seq analysis

All public RNA-seq datasets used in this study were downloaded from the Sequence Read Archive (SRA; http://www.ncbi.nlm.nih.gov/sra/) database (Additional file 2: Table S1). For T-DNAreader analysis, the paired-end FASTQ files for each strand were independently aligned to the *Arabidopsis* (TAIR10), rice (IRGSPv1.0), *M. polymorpha* (MpTak1v5.1), poplar (*P. trichocarpa* 533-v4.0), *Z. tritici* (IPO323v2.0), *V. dahliae* (VdLs.17), or *A. chrysogenum* (*ATCC 11550*) genomes. To run T-LOC, the sequencing reads were mapped to TAIR10 or the corresponding T-DNA sequences (pROK2 for SALK lines, pAC161 for GABI-Kat lines, and pCSA110 or pDAP101 for SAIL lines). STAR (v2.7.10a) [42] was employed to align the RNA-seq reads with the parameters of “--pOverlapNbasesMin 12 --peOverlapMMp 0.1 --twopassMode Basic --alignIntronMax 30,000 --outFilterMatchNminOverLread 0.33–0.67 --outFilterScoreMinOverLread 0.33–0.67 --outFilterMismatchNoverLmax 0.33–0.67.” Bigwig files were generated to visualize the RNA-seq coverage using BamCoverage from DeepTools [126]. Transcript abundance was quantified using RSEM (v1.3.1) [127].

### Benchmark with T-LOC and TDNAscan

Given that TDNAscan supports only paired-end reads, RNA-seq datasets with paired-end reads were used for the comparison. T-LOC supports both FASTQ and BAM formats as inputs; therefore, we ran T-LOC with both FASTQ files that utilize BWA [44] and BAM files produced using STAR [42]. Furthermore, as T-LOC supports multiple control data, we ran T-LOC twice using the default parameters: once with multiple control BAM files (T-LOC-multi) and once with a single control BAM file (T-LOC-single). To run TDNAscan, raw sequencing reads were trimmed using Trim Galore [128, 129] with the parameters of “-q 20 --phred33.” TDNAscan was implemented with default parameters, and the identified TISs were filtered using the insertion frequency threshold of 0.05 (TDNAscan-0.05) or 1.0 (TDNAscan-1.0).

The sensitivity and precision of each TIS-identifying tool were calculated as below. TP is the count of annotated and identified TISs, FN is the count of annotated but not identified TISs, and FP is the count of identified TISs that have not been previously annotated.

$$\mathrm{Sensitivity}=\frac{\mathrm{TP}}{\mathrm{TP}+\mathrm{FN}},\mathrm{Precision}=\frac{\mathrm{TP}}{\mathrm{TP}+\mathrm{FP}},$$

$$\mathrm F1\;\mathrm{score}=2\times\frac{\mathrm{Sensitivity}\;\times\;\mathrm{Precision}}{\mathrm{Sensitivity}\;+\;\mathrm{Precision}}$$

To measure the runtime of T-DNAreader, TDNAscan, and T-LOC, we used RNA-seq data of SRR14850977, which contains more than 60 million reads. A random subsampling of sequencing reads was performed using seqtk (v1.4) [130] with a random state value of 42. T-LOC was executed using pre-aligned BAM files excluding the initial alignment time, and the process that generates additional coverage track files was excluded from the runtime estimation. As TDNAscan accepts only FASTQ files as inputs, the initial alignment time was excluded from the runtime estimation. The runtime for each tool was measured three times to ensure the consistency of the results.

### Hi-C data processing and EagleC analysis

Raw Hi-C reads were downloaded from the SRA database. Juicer (v1.22) [131] was employed to process the Hi-C reads with the reference genome of TAIR10. The aligned reads were filtered with a mapping quality cutoff value of 30. The Knight-Ruiz (KR)-normalized contact matrices were used for the visualization. To predict chromosomal rearrangements, EagleC (v0.1.9) [101] was employed with the parameters “--balance-type ICE --output-format full -C #” at a 10-kb resolution.

### WGS analysis

For the WGS analysis in rice and *Arabidopsis*, raw sequencing reads were downloaded from the SRA database (Additional file 2: Table S1). Sequencing reads were aligned to the *Arabidopsis* (TAIR10), rice (IRGSPv1.0), or *Z. tritici* (IPO323v2.0) genomes, using Bowtie2 [43] in local mode with default parameters. Similar to the RNA-seq analysis, paired-end reads were treated as single-end reads and aligned separately using the “-U” option. The output BAM files were further processed with a mapping quality threshold of 30. Bigwig files were generated to visualize the WGS coverage using BamCoverage from DeepTools [126]. In the rice analysis, the commonly identified regions across all samples were defined as blacklist regions (Additional file 2: Table S11).

### Genotyping

To validate the newly identified TISs by T-DNAreader, forward (F1) and reverse (R1) primers were designed to bind genomic regions encompassing the identified TISs. The B1 primers (B1) were designed to specifically bind to T-DNA. The primer pair of F1-R1 was used to amplify genomic fragments, while the primer pairs of F1-B1 and B1-R1 were used for the amplification of chimeric fragments containing the TISs. The amplified products were purified and sequenced. The primers used in this study are listed in Additional file 2: Table S12.

### Data visualization

Normalized Hi-C contact maps were visualized using JuiceBox [131]. All heatmaps and one-dimensional plots were generated with matplotlib [132] and seaborn [133].

## Supplementary Information

Additional file 1: Supplementary Figures S1-S10.

Additional file 2: Supplementary Tables S1-S12.

## Data Availability

All public datasets used in this study are listed in Additional file 2: Table S1 [47-87, 90-100, 102-124]. The raw data and all implemented codes used in this study have been deposited in Zenodo (DOI: 10.5281/zenodo.15300393) [134]. The source code of T-DNAreader is freely available on GitHub (https://github.com/CDL-HongwooLee/TDNAreader) [135] under a GNU GPL-3.0 license.
