# Supplementary material for: T-DNAreader: fast and precise identification of T-DNA insertion sites in plant genomes using RNA sequencing data
Source: Genome Biol. 2025 Jul 10;26:199. doi: 10.1186/s13059-025-03655-x (PMC12243177; doi:10.1186/s13059-025-03655-x)
Supplement: Supplementary file 1 — Additional file 1: Supplementary Figures S1-S10. [file 13059_2025_3655_MOESM1_ESM.pdf]

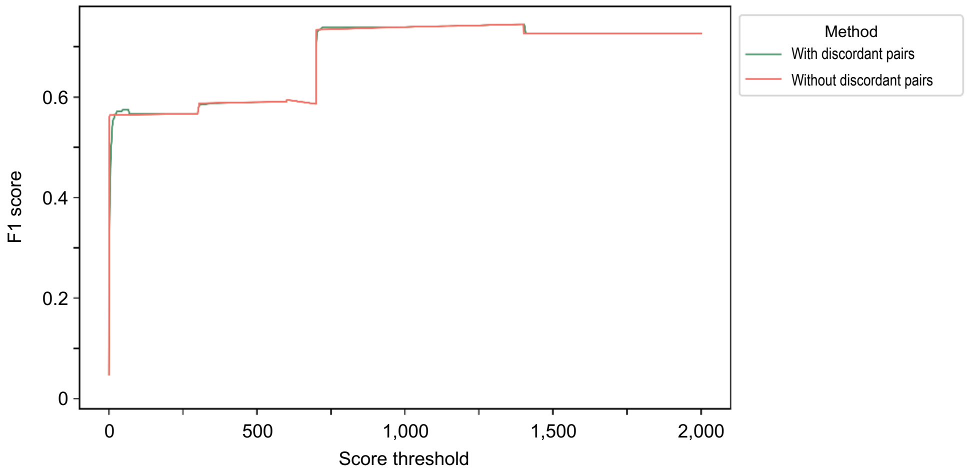

Fig S1. Comparison of T-DNAreader performance with or without using discordant pairs. Distribution of F1 scores across different T-DNAreader score threshold is shown. Scores are calculated with or without using discordant pairs (group 5). For the model including discordant pairs, a weight value of 1 was applied for score calculation.

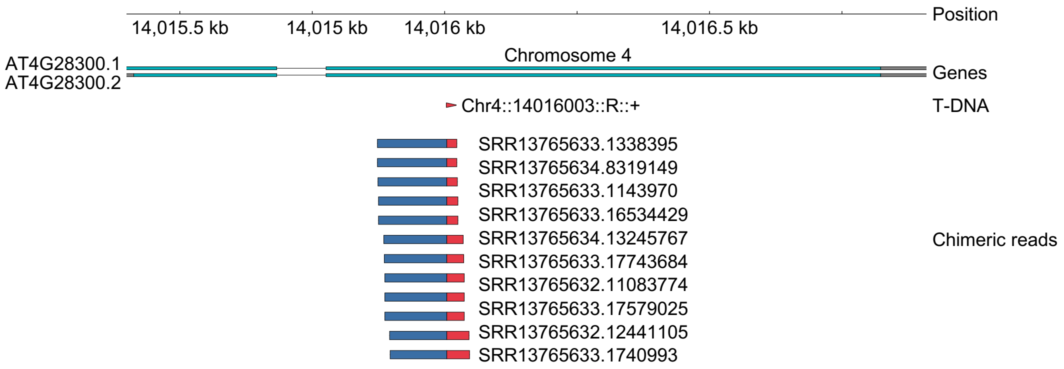

Fig S2. Example output from T-DNAreader showing TISs with supporting reads.
T-DNAreader plots genes and identified TISs according to their orientation. Blue boxes represent read segments aligned to the reference genome, whereas red boxes represent segments aligned to the T-DNA sequence.

Chr1:18979275-TDNA

>SRR14850978.23897795
CAATTGCAGGAAAAGTAGCGGATTCTCGCGTGGTGCATCCATGTATCGTGGAGTAACAAGGCATCATCAACATGGAAGATGGCAAGCAAGGATCG
GCCGAGTTG CTTACATAATTCACTCAAATGCTAGGCAGTCTGTCAACTCGGCGTCA
>AT1G51190
CAATTGCAGGAAAAGTAGCGGATTCTCGCGTGGTGCATCCATGTATCGTGGAGTAACAAGGCATCATCAACATGGAAGATGGCAAGCAAGGATCG
GCCGAGTTG **CT**GGAAACAAAGATCTCTACTTGGGAACATTCA
>pAC161
AATTCAGTGTAGCCCATT**AC** CTTACATAATTCACTCAAATGCTAGGCAGTCTGTCAACTCGGCGTCAATTTG

Chr1:18979291-TDNA

>SRR14850977.2319171
GCGGATTCTCGCGTGGTGCATCCATGTATCGTGGAGTAACAAGGCATCATCAACATGGAAGATGGCAAGCAAGGATCGGCCGAGTTGCTGGAAA
CAAAGATCT CTTACATAATTCACTCAAATGCTAGGCAGTCTGTCAACTCGGCGTCAA
>AT1G51190
GCGGATTCTCGCGTGGTGCATCCATGTATCGTGGAGTAACAAGGCATCATCAACATGGAAGATGGCAAGCAAGGATCGGCCGAGTTGCTGGAAA
CAAAGATCT **CT**ACTTGGGAACATTCA
>pAC161
AATTCAGTGTAGCCCATT**AC** CTTACATAATTCACTCAAATGCTAGGCAGTCTGTCAACTCGGCGTCAATTTG

Chr1:18979571-TDNA

>SRR14850977.60730996
TGTATCGTGGAGTAACAAGGCATCATCAACATGGAAGATGGCAAGCAAGGATCGGCCGAGTTGCTGGAAACAAAGATCTCTACTTGGGAACATTC
A GCAGTCTGTCAACTCGGCGTCAATTTGTCGGCCACTATACGATAGTTGCGCAAAT
>AT1G51190
TGTATCGTGGAGTAACAAGGCATCATCAACATGGAAGATGGCAAGCAAGGATCGGCCGAGTTGCTGGAAACAAAGATCTCTACTTGGGAACATTC
A **GT**AAGAATAAATATTAA
>pAC161
CACTCAAATGCT**AG** GCAGTCTGTCAACTCGGCGTCAATTTGTCGGCCACTATACGATAGTTGCGCAAAT

Chr3:7302676-TDNA

>SRR14850978.50595879
GGGAACTTTTAGCACTGAGGAAGAAGCAGCAGAAGCTTACGATATAGCTGCAATAAAGTTTAGAGGACTTAATGCAGTGACCAACTTCGAGATCAA
CCG TGAGACGGGCAACAGCTGATTGCCCTTCACCGCCTGGCCCTGAGAGAGTTGC
>AT3G20840
GCACTGAGGAAGAAGCAGCAGAAGCTTACGATATAGCTGCAATAAAGTTTAGAGGACTTAATGCAGTGACCAACTTCGAGATCAACCG
GTACGACGTGAAAGCCATT
>pROK2
GTTTTTCTTTTCACC**AG** TGAGACGGGCAACAGCTGATTGCCCTTCACCGCCTGGCCCTGAGAGAGTTGC

Fig S3. Examples of T-DNAreader-identified T-DNA insertion sites (TISs) from the *plt1-21 plt2-21* mutant.
The RNA-seq chimeric reads containing both plant genomic and T-DNA sequences are shown. Original plant genome and T-DNA sequences near the TISs are also shown for comparison. The genomic and T-DNA sequences that overlap with the chimeric reads are colored blue and red, respectively. Splicing junction sequences of GT or AG are highlighted in green.

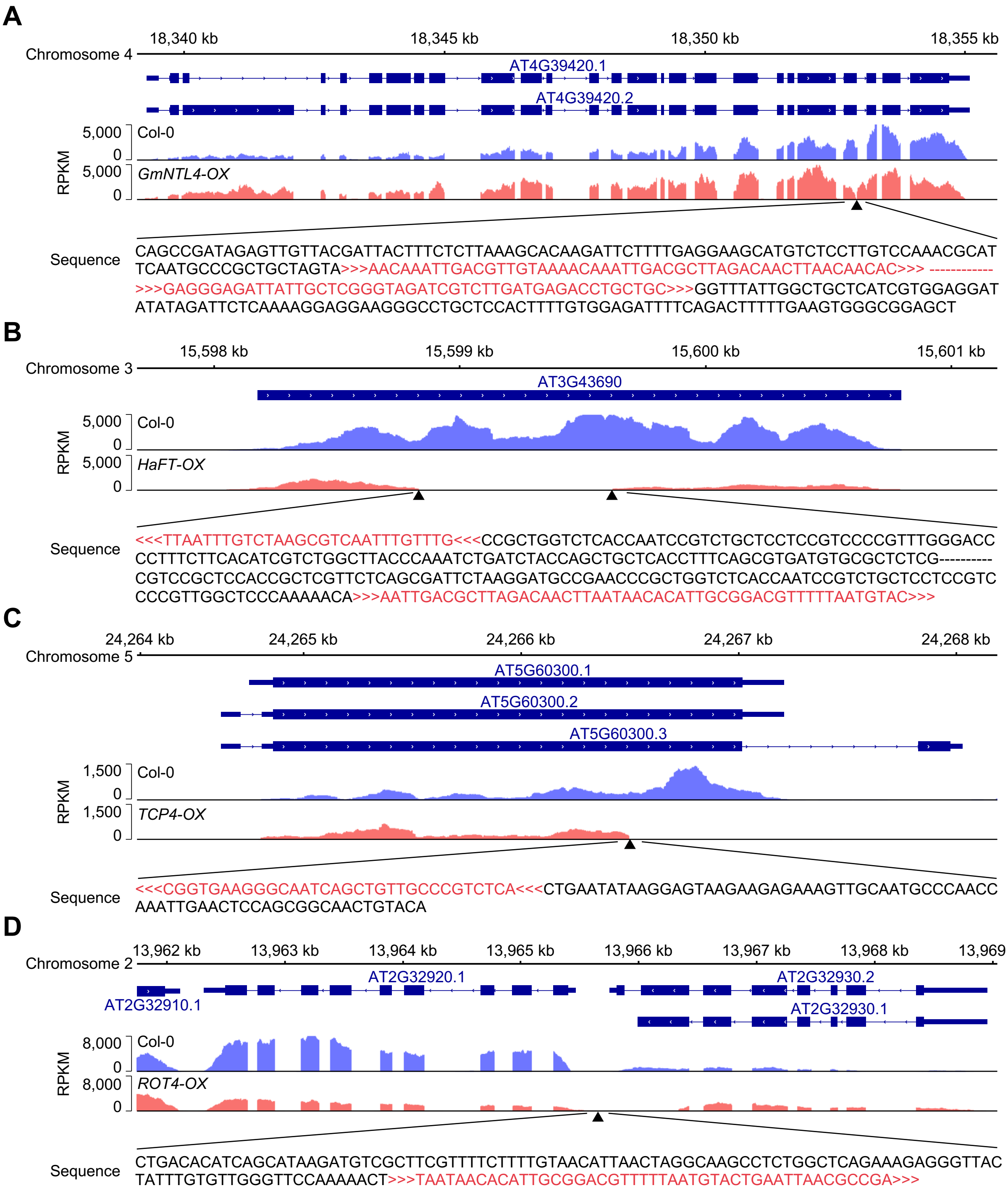

Fig S4. Examples of T-DNAreader-identified T-DNA insertion sites (TISs) from RNA-seq data of transgenic overexpression (OX) lines.

(A–D) RNA-seq coverage plots at T-DNAreader-identified TISs in the wild-type (Col-0) and transgenic overexpression lines. The RNA-seq coverage was calculated as the number of reads per kilobase of transcript per million mapped reads (RPKM). The black triangles indicate the T-DNAreader-identified TISs. The T-DNA sequences are colored red, and the orientation of the T-DNA insertion is indicated.

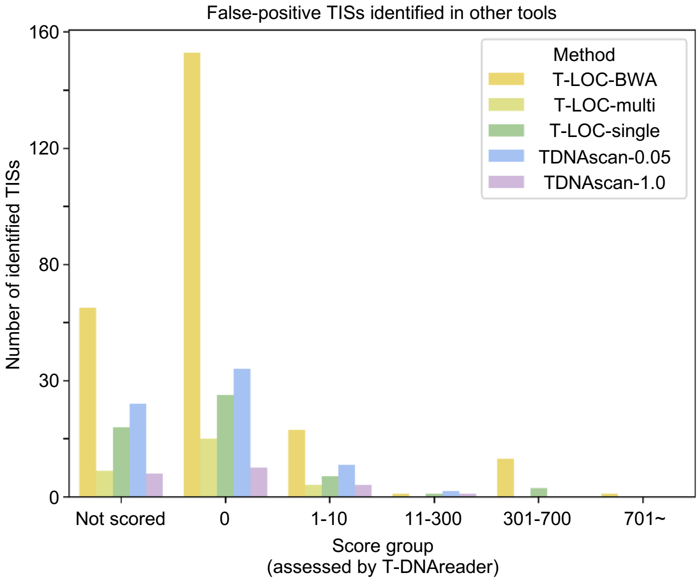

Fig S5. Score distribution of false-positive TISs from existing tools.
The score distribution of false-positive TISs, which do not exhibit changes in RNA-seq signals around the identified sites, is shown. TISs that T-DNAreader could not detect even without applying a score cutoff are labeled as "Not scored". The false-positive TISs are divided into five groups according to their scores.

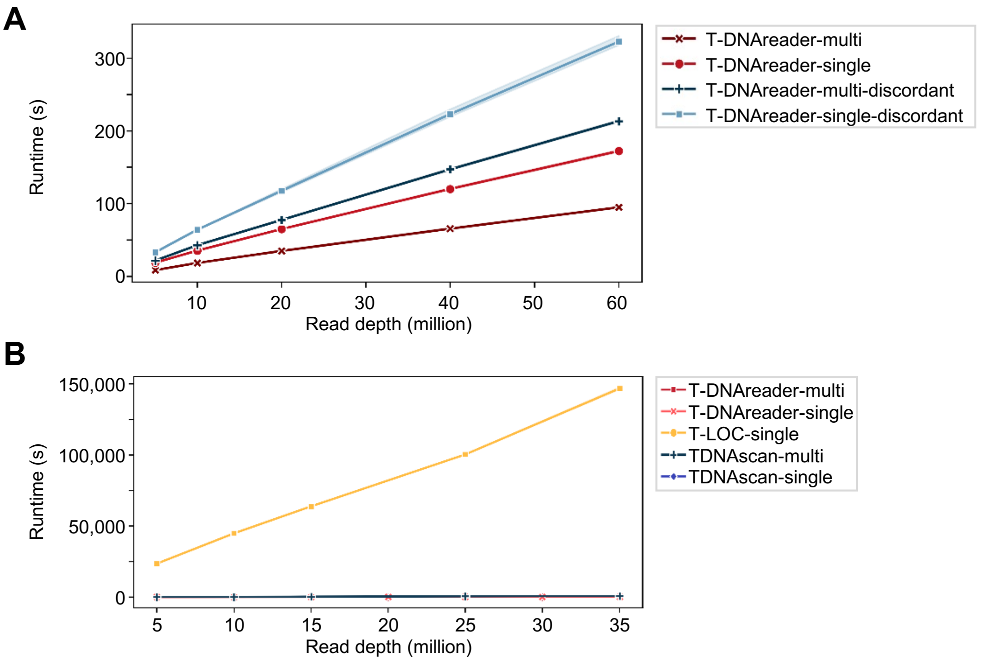

Fig S6. Runtime comparison between T-DNAreader, T-LOC, and TDNAscan.
(**A**) Runtime comparison of T-DNAreader with or without the '--discordant' option, which utilizes discordant paired-end read information for TIS identification. T-DNAreader was tested with a single thread (T-DNAreader-single) and two threads (T-DNAreader-multi). (**B**) Runtime comparison between T-DNAreader, T-LOC, and TDNAscan using subsampled RNA-seq data from SRR19019339. T-LOC was tested in single-control mode (T-LOC-single), while TDNAscan was run with a single thread (TDNAscan-single) and with eight threads (TDNAscan-multi).

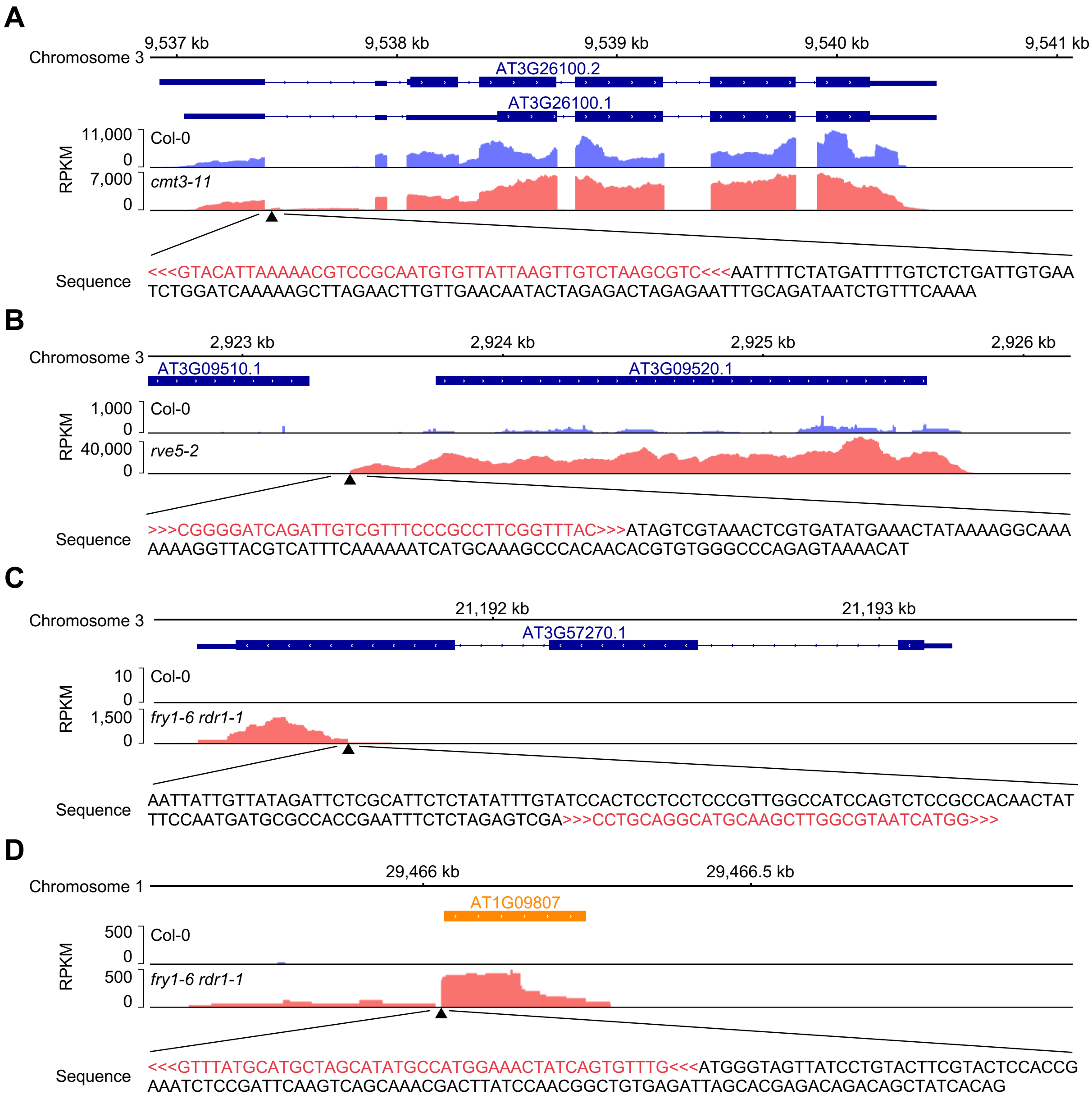

Fig S7. Continued

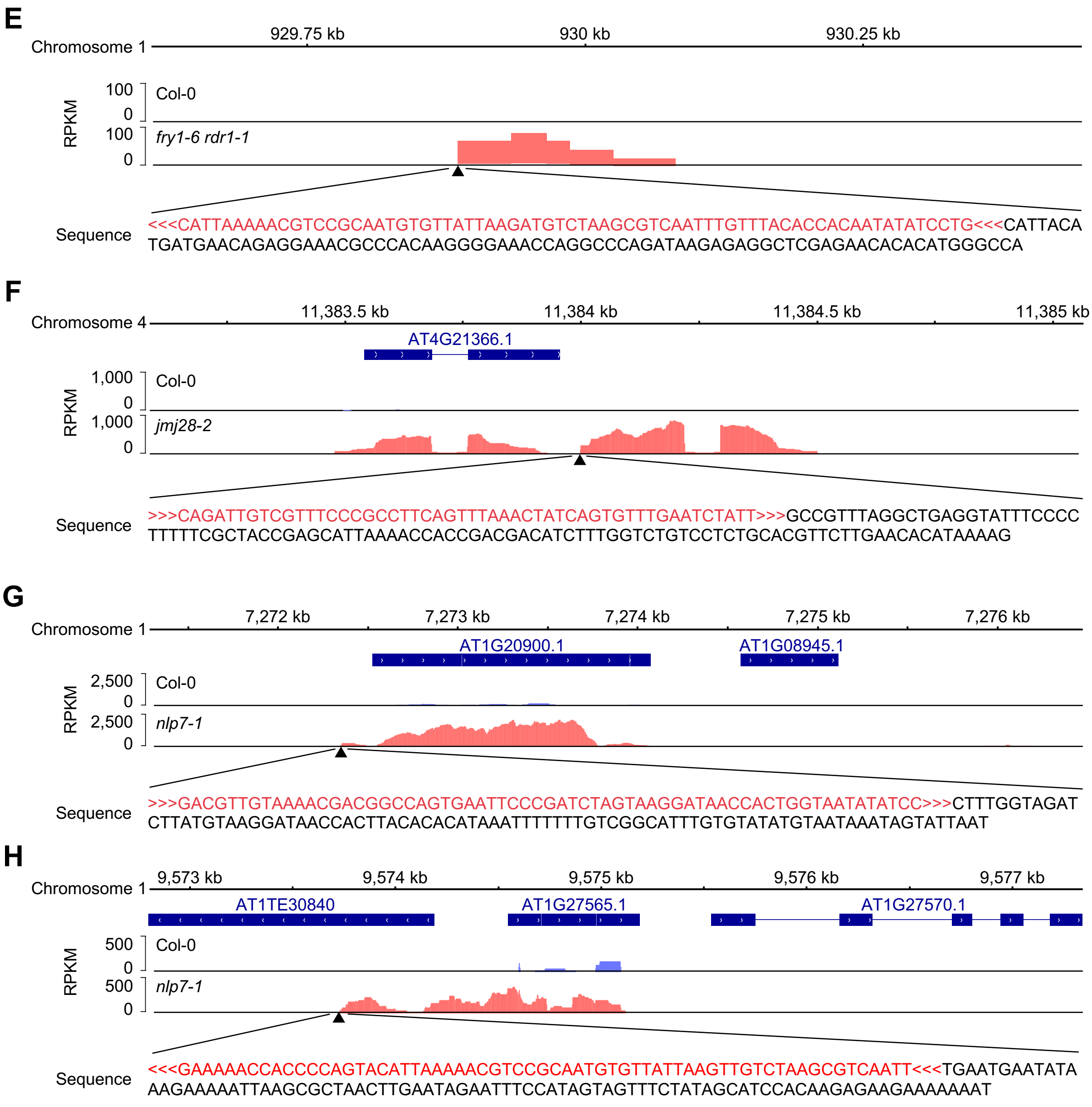

Fig S7. Misregulation of RNA splicing or gene transcription at the previously uncharacterized T-DNA insertion sites (TISs).

(A–H) RNA-seq coverage plots at previously unannotated TISs in wild-type (Col-0) and T-DNA insertion mutant plants. RNA-seq coverage was calculated as the number of reads per kilobase of transcript per million mapped reads (RPKM). Black triangles indicate TISs identified by T-DNAreader. The T-DNA sequences are shown in red, with insertion orientation indicated.

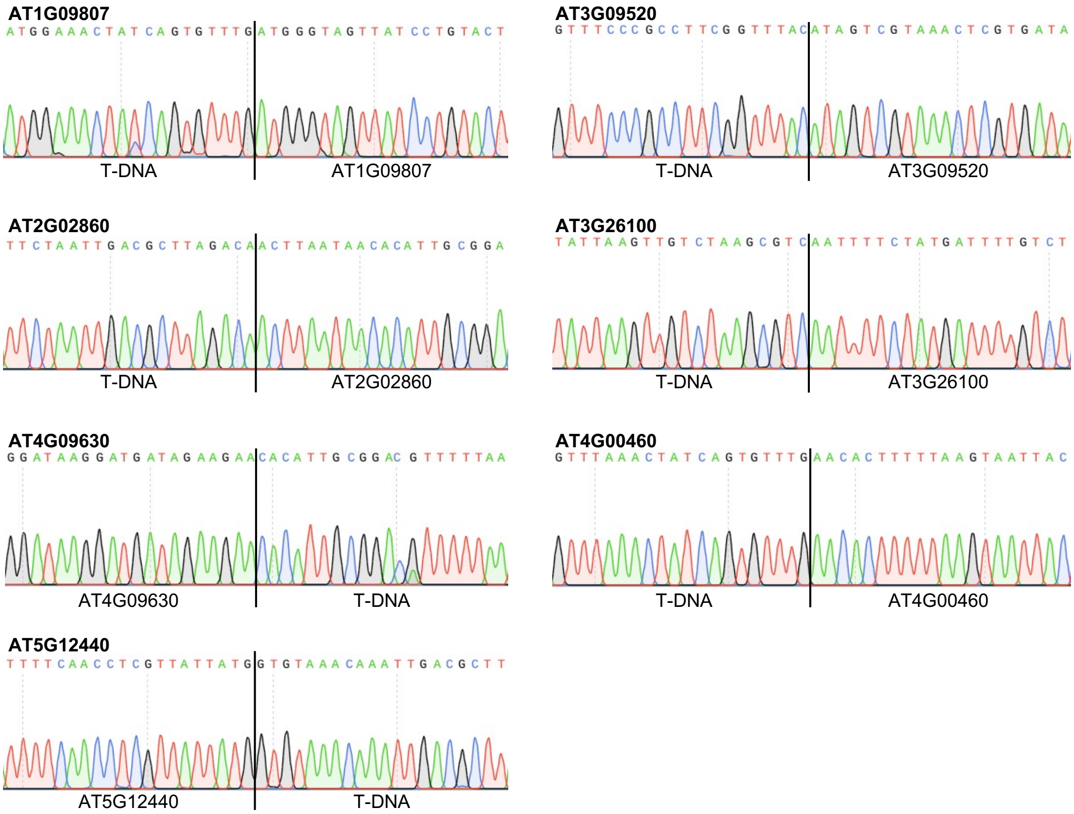

Fig S8. Sequencing results of amplified chimeric fragments at the previously unannotated T-DNA insertion sites (TISs).

The Sanger sequences of the amplified chimeric fragments at the previously unannotated TISs in the *Arabidopsis* genes *AT1G09807, AT2G02860, AT3G09520, AT3G26100, AT4G00460, AT4G09630,* and *AT5G12440* are displayed.

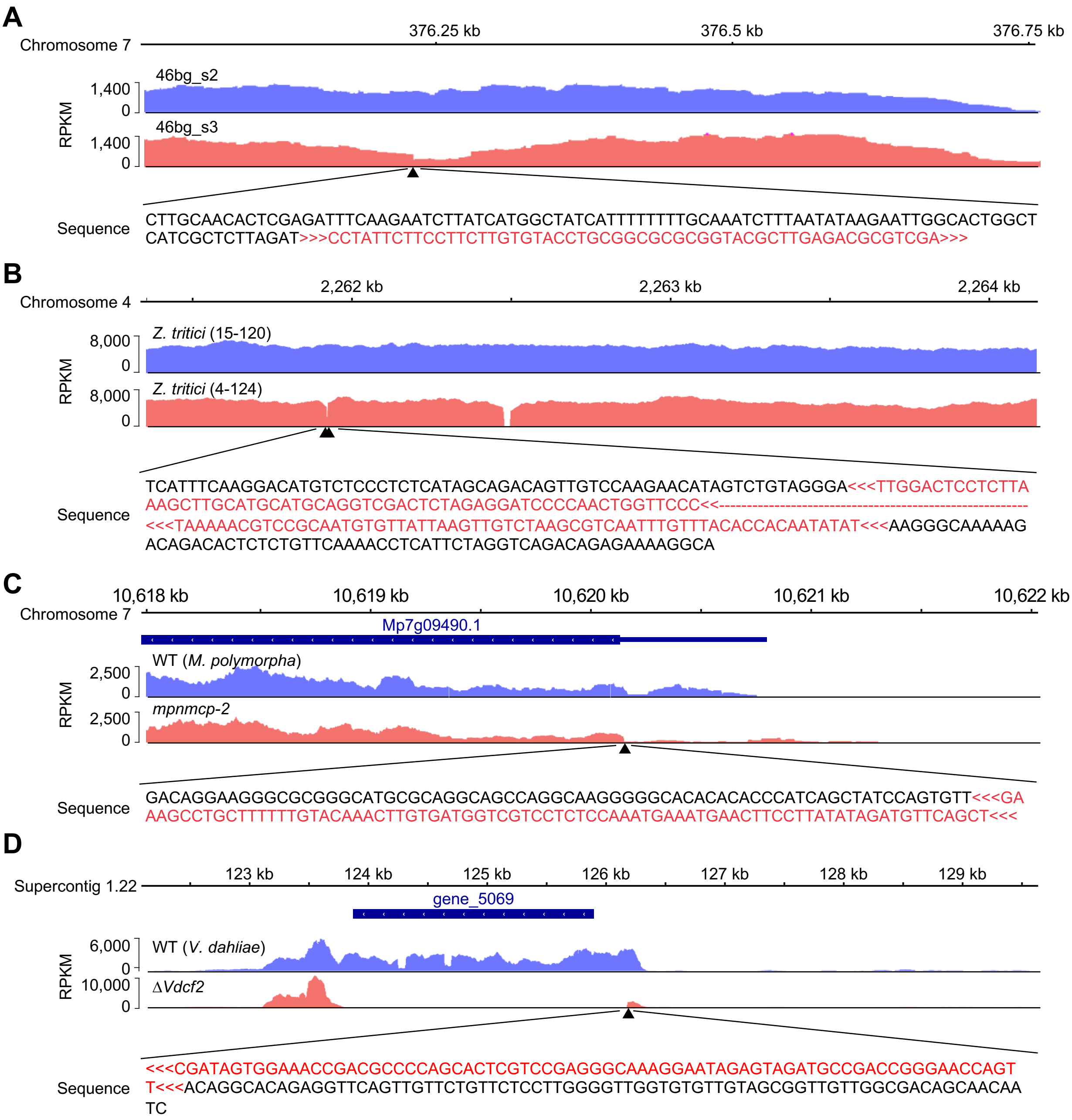

Fig S9. Examples of T-DNAreader-identified T-DNA insertion sites (TISs) in other species. **(A–D)** Coverage profiles at TISs identified in rice (**A**), *Z. tritici* (**B**), *M. polymorpha* (**C**), and *V. dahliae* (**D**). WGS (**A** and **B**) and RNA-seq (**C** and **D**) data were used for TIS identification. The coverage value was calculated as the number of reads per kilobase of transcript per million mapped reads (RPKM). The black triangles indicate the T-DNAreader-identified TISs. The T-DNA sequences are colored red, and the insertion orientation is indicated.

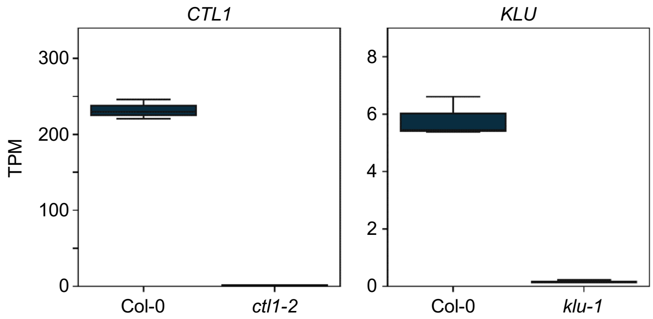

Fig S10. Expression levels of the *CTL1* and *KLU* genes in T-DNA insertion mutants.
Expression levels of *CTL1* and *KLU* in wild-type (Col-0) plants and the T-DNA insertion mutants *ctl1-2* and *klu-1*. The *ctl1-2* and *klu-1* mutants contain T-DNA insertions within their promoter and 5′-UTR regions, respectively. Expression levels were quantified as Log_2(transcripts per million (TPM) + 1).
